# Treatment of anastomotic leakage following Ivor Lewis esophagectomy—10 year experience from a Nordic center

**DOI:** 10.1093/dote/doae040

**Published:** 2024-05-14

**Authors:** Tobias Hauge, Thomas Dretvik, Egil Johnson, Tom Mala

**Affiliations:** Department of Pediatric and Gastrointestinal Surgery, Oslo University Hospital, Ullevål, Norway; Department of Pediatric and Gastrointestinal Surgery, Oslo University Hospital, Ullevål, Norway; Department of Pediatric and Gastrointestinal Surgery, Oslo University Hospital, Ullevål, Norway; Institute of Clinical Medicine, University of Oslo, Norway; Department of Pediatric and Gastrointestinal Surgery, Oslo University Hospital, Ullevål, Norway; Institute of Clinical Medicine, University of Oslo, Norway

**Keywords:** anastomotic leakage, bronchial fistula, esophagectomy, negative-pressure wound therapy, stent

## Abstract

Anastomotic leakage (AL) is a dreaded complication following esophageal resection. No clear consensus exist for the optimal handling of this severe complication. The aim of this study was to describe the treatment outcome following AL. We conducted a retrospective cross-sectional study including all patients with AL operated with Ivor Lewis esophagectomy from 2010 to 2021 at Oslo University Hospital, Norway. 74/526 (14%) patients had AL. Patient outcomes were analyzed and categorized according to main AL treatment strategy; stent (54%), endoscopic vacuum therapy and stent (EVT + stent) (19%), nasogastric tube and antibiotics (conservative) (16%), EVT (8%) and by other endoscopic means (other) (3%). One patient had surgical debridement of the chest cavity. In 66 patients (89%), the perforation healed after median 27 (range: 4–174) days. Airway fistulation was observed in 11 patients (15%). Leak severity (ECCG) was associated with development of airway fistula (*P* = 0.03). The median hospital and intensive care unit stays were 30 (range: 12–285) and 9 (range: 0–60) days. The 90-days mortality among patients with AL was 5% and at follow up, 13% of all deaths were related to AL. AL closure rates were comparable across the groups, but longer in the EVT + stent group (55 days vs. 29.5 days, *P* = 0.04). Thirty-two percent developed a symptomatic anastomotic stricture within 12 months. Conclusion: The majority of AL can be treated endoscopically with preservation of the conduit and the anastomosis. We observed a high number of AL-associated airway fistulas.

## INTRODUCTION

Intrathoracic esophageal anastomotic leaks (ALs) continue to be a serious surgical complication, causing morbidity, mortality and a significant amount of hospital recourses expenditure. AL may also adversely affect oncological outcome.^1^

The incidence of AL after esophagectomy varies from 3 to 30% across reports. AL is a potential life-threatening condition due to septicemia, mediastinitis, multiorgan failure, and fistulation into neighboring structures, in particular the airways. A variety of treatment options are available, ranging from extensive surgery, less invasive endoscopic or radiological procedures, to drainage and antibiotics only. No general consensus or sufficient evidence to support a particular management strategy exist.^2^ The treatment strategies varies according to local expertise, time elapsed since primary surgery, location of the leakage, duration and extent of contamination, viability of the conduit, and the patient’s overall condition.^3^

Self-expanding metal stents (SEMS) has been the standard treatment of AL at many centers, often combined with percutaneous drainage of mediastinal fluids collections and abscesses.^4^^,^^5^ However, endoscopic vacuum therapy (EVT) has been introduced in recent years as a treatment option and may confer advantages with regard to healing rates.^6–9^ By removing necrotic debris and pus, EVT with its persistent negative pressure is theorized to preventing further spread of the contamination and to promote tissue granulation.^8^ EVT may also be used as part of a step-up procedure with subsequent stenting following removal of debris.

Improved evidence and knowledge related to handling of esophageal AL are needed. We aimed to report our single center experience during a 10-year period with esophageal AL. The main aim was to describe AL closure rates by different treatment modalities and the secondary aim was to examine the extent of specific treatment associated resources.

## METHODS

This is a retrospective cross-sectional study, encompassing all patients operated for esophageal cancer with Ivor Lewis esophagectomy with AL at Oslo University Hospital, Norway from January 2010 to December 2021. The patients were followed for a minimum of 12 months postoperative. Oslo University Hospital is a regional referral center for esophageal cancer in South-East Norway with currently 3.1 million inhabitants. At our center, three to four upper GI surgeons operate some 60 esophagectomies annually, more than 95% being Ivor Lewis esophagectomies.

Preoperatively, patients routinely underwent spirometry. The forced expiratory volume in the first second (FEV1) and the diffusing capacity of the lung for carbon monoxide (DLCO) were registered.

Until June 2013, all surgeries were conducted using the hybrid minimally invasive esophagectomy (HMIE) technique, which involved laparoscopy and right-sided thoracotomy. From June 2013, total minimally invasive esophagectomy by laparoscopy and thoracoscopy (TMIE) was performed. Patients operated with other surgical techniques, non-malignant cases or palliative resections were excluded. None of the procedures were robotically assisted. The anastomosis was typically made using a circular stapler in an end-side fashion with transorally positioning of the orvil (EEA Circular stapler 25 mm). A standard 2-field lymphadenectomy was conducted. Thoracic drains were routinely positioned toward the anastomosis and lower parts of the thoracic cavity.

Postoperatively, the patients received a nasogastric tube and was held on nil per os the first 3 days. If no sign of retention or AL, the tube was removed and they were allowed to drink fluids before gradually introducing more solid food. All patients remained at the postoperative ward for at least 3 days before transferal to the regular ward. In the early study period, we did not routinely evaluate all esophagogastrostomies, but on clinical suspicion at the discretion of the attending surgeon. From October 2017 until July 2020, all patients were scheduled for an upper endoscopy at postoperative day 3. Enhanced Recovery Program (ERP), with a standardized perioperative follow-up regime, was introduced in March 2016.

Traditionally, we have treated AL non-operatively with covered or semi-covered metal stents. EVT was introduced in 2015 using a homemade system; we suture a polyurethane sponge (V.A.C.® Granufoam, KCI), approximately 7 cm long, to the distal part of a dual-flow nasogastric tube (14 Fr). The sponge is placed endoscopically guided under deep sedation and is connected to a vacuum pump with a negative pressure of 125 mmHg with continuous suction. In most cases, the sponge was placed in the esophageal lumen (intraluminal) centered at the defect, but in a few cases the sponge was placed into the perforation (extraluminal). The sponge was changed every 3–5 days, while the stent remains for about 2–3 weeks with some individual variation. Patients receiving EVT and stent treatment were handled on the regular ward or at the postoperative/intensive care unit (ICU) depending on the need for organ support and surveillance. The preferred treatment modality was based on a case-by-case evaluation by the surgical team in cooperation with the gastroenterologists. Small leakages were typically handled conservatively with nil per os and a nasogastric tube. All patients were given broad spectrum iv-antibiotics and pleural fluid, mediastinal abscesses and/or empyema were drained percutaneously.

Data were retrospectively registered (June 2022) using the patient administration system and a local patient registry of all patients receiving esophageal cancer surgery. The use of patient data and the registry was approved by the institutional Data Protection Officer. The Regional Ethical Committee waived the need for Ethical approval of the study. The study was reported using the STROBE statement.^10^

### Definitions

AL was defined according to the Esophageal Complications Consensus group (ECCG) criteria as a ‘full thickness gastrointestinal defect involving esophagus, anastomosis, staple line, or conduit irrespective of presentation or methods of identification’.^11^ The leakages were subclassified based on the treatment required, ranging from ECCG type I to III. A type I defect is a defect that requires no change in therapy or is treated medically, a type II defect requires non-surgical intervention (endoscopically or radiological treatment), while a defect type III requires surgical therapy.^11^ We defined a healed defect when there was no visual defect on endoscopy and/or no signs of contrast leakage on CT/contrast examinations. A post-leakage stenosis was defined as a symptomatic anastomotic stenosis needing treatment.

For assessment of preoperative comorbidity, we used the American Society of Anesthesiologists (ASA) physical status classification system, ranging from 1 (healthy person) to 6 (a declared brain-dead person undergoing organ donation) and the Clinical Frailty scale, ranging from 1 (very fit) to 7 (severely frail—completely dependent on others for the daily activities of living).^12^ The Union for International Cancer Control TNM version 7 was used to classify all tumors. The postoperative TNM stage is reported, except for a minority of patients that did not have any residual tumor left in in the operation specimen in which the preoperative clinical TNM stage was used (cTNM).

Postoperative complication profiles were assessed using the Clavien Dindo (CD) Classification system, ranging from grade I (minimal complication) to grade V (death). Only grade IIIa (requiring surgical, endoscopic, or radiological procedures with therapeutic interventions without the use of general anesthesia) or more advanced complications were registered.

### Statistics

Continuous data are presented as mean and/or median as appropriate. When comparing outcome between different treatment modalities, the ‘stent group’ was set as a reference population and *t*-test and proportion test were used to calculate *P*-value. *P*-values <0.05 were considered statistically significant, but was not calculated for the group ‘other’ due to only two included participants. Microsoft Excel version 16 and STATA/SE version 17.0 were used for statistical analysis.

## RESULTS

**Fig. 1:**
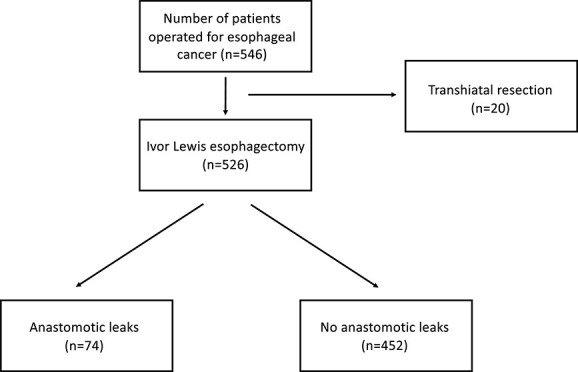
Flowchart of all patients operated for esophageal cancer at Oslo University Hospital from 2010 to 2021.

A total of 526 patients were operated with Ivor Lewis esophagectomy, of which 112 patients (21%) were operated with HMIE and 414 (79%) with TMIE. Seventy-four of these (14%) had AL and were included in the study (Fig. 1). Two patients had a tumor stage of T1a (3%), 11 T1b (15%), 17 T2 (23%), 40 T3 (54%) and 4 patients had T4a (5%). Forty-five patients did not have any lymph node metastasis (61%), 14 had N1 disease (19%), 9 had N2 (12%) and 6 patients had N3 (8%). None of the patients had distant metastasis. Patient characteristics, the use of neoadjuvant treatment, spirometry evaluation, tumor histology, and technical details regarding the anastomosis are described in Table 1.

**Table 1:** Patient characteristics and treatment aspects in 74 patients treated for AL after Ivor Lewis esophagectomy at Oslo University Hospital from 2010 to 2021

	**Descriptive data and histology.**					
	**All patients (*n* = 74)**	**EVT (*n* = 6)**	**Stent (*n* = 40)**	**EVT + stent (*n* = 14)**	**Conservative (*n* = 12)**	**Other (*n* = 2)**
Age (median)	66	72	66	68.5	66	59
Sex (men)	63	6	33	13	10	1
ASA (median)	2	2	2	2	3	2
COPD	13 (18%)	0	6 (15%)	4 (29)	3 (25%)	0
Frailty score (median)	3	3	3	2	3	3
Diabetes	12 (16%)	2 (33%)	7 (18%)	1 (7%)	2 (17%)	0
Smoking	29 (39%)	1 (17%)	17 (43%)	4 (29%)	6 (50%)	1 (50%)
HMIE	4 (5%)	0	4 (10%)	0	0	0
TMIE	70 (95%)	6 (100%)	36 (90%)	14 (100%)	12 (100%)	2 (100%)
Neoadjuvant radiochemotherapy	27 (37%)	1 (17%)	18 (45%)	5 (36%)	3 (25%)	0
Neoadjuvant chemotherapy	25 (34%)	1 (17%)	13 (33%)	5 (36%)	4 (33%)	2 (100%)
Stapled end-side anastomosis (circular)	72 (97%)	6 (100%)	38 (95%)	14 (100%)	12 (100%)	2 (100%)
Stapled side-side anastomosis (straight)	2 (3%)	0	2 (5%)	0	0	0
FEV1 (% Predicted)	67.5	83.3	53.2	88.5	70.9	87.0
DLCO (% Predicted)	66.0	81.6	48.1	96.6	71.0	74.0
**Histology**						
*Adenocarcinoma*	61 (82%)	5 (83%)	34 (85%)	12 (86%)	8 (67%)	2 (100%)
*Squamous cell carcinoma*	10 (14%)	0	6 (15%)	1 (7%)	3 (25%)	0
*Other*	3 (4%)	1 (17%)	0	1 (7%)	1 (8%)	0

ASA, American Society of Anesthesiologists Physical Status; COPD, chronic obstructive pulmonary disease; HMIE, hybrid minimally invasive esophagectomy; TMIE, totally minimally invasive esophagectomy; EVT, endoscopic vacuum therapy

### AL management and outcome

**Fig. 2:**
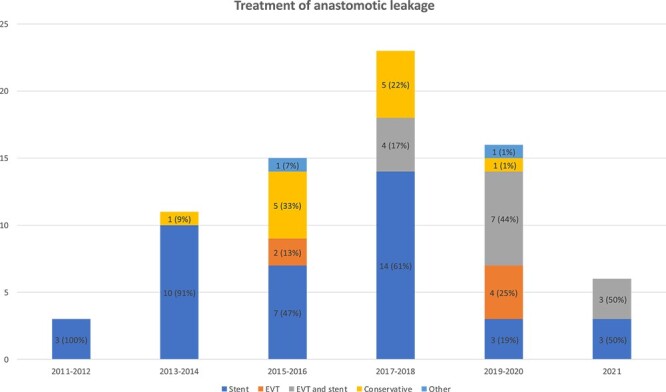
Initial treatment and number of ALs after Ivor Lewis esophagectomy per two years during 2010–2021.

The AL was initially diagnosed at a median of 8 (range 1–78) days after the esophagectomy by CT in 45 patients (61%), upper endoscopy in 24 patients (32%) and by esophagogram with peroral contrast in 5 patients (7%) (Table 2). The main AL treatment approach was stent in 40 patients (54%), a combination of stent and EVT in 14 patients (19%), conservative treatment in 12 patients (16%) and EVT in 6 patients (8%). Two patients (3%) were treated by other endoscopic means with glue, mesh, and clips (*n* = 1) or with a flat drain placed through the perforation and into a mediastinal abscess (*n* = 1), respectively. The number of AL and the treatment chosen varied over time (Fig. 2).

**Table 2:** Classification of ALs and initial diagnostic modality in 74 patients after Ivor Lewis esophagectomy

	**Classification of leakage and diagnostics. Number of patients.**					
	**All patients (*n* = 74)**	**EVT (*n* = 6)**	**Stent (*n* = 40)**	**EVT + stent (*n* = 14)**	**Conservative (*n* = 12)**	**Other (*n* = 2)**
Day of leak (median/range)	8 (1–78)	9 (1–12)	8 (1–53)	8 (2–78)	7 (4–16)	13 (10–16)
**Classification of leakage (ECCG)**						
*1*	9 (12%)	0	0	0	9 (75%)	0
*2*	64 (86%)	6 (100%)	39 (98%)	14 (100%)	3 (25%)	2 (100%)
*3*	1 (14%)	0	1 (2%)	0	0	0
**Diagnosis made by**						
*Upper endoscopy*	24 (32%)	0	16 (40%)	2 (14%)	4 (33%)	2 (100%)
*CT*	45 (61%)	5 (83%)	22 (55%)	11 (79%)	7 (58%)	0
*Esophagram with PO contrast*	5 (7%)	1 (17%)	2 (5%)	1 (7%)	1 (8%)	0

CT, computer tomography; EVT, endoscopic vacuum therapy

From October 2017 until July 2020, all patients underwent an upper endoscopy on postoperative day 3. During this period, 24 out of 138 patients were diagnosed with AL (17.4%). This rate was comparable to the AL rate before and after the period with routine use of upper endoscopy at day 3 (12.9%, *P* = 0.19).

In total, the AL was treated conservatively (ECCG I) in nine patients (12%), endoscopically and/or by radiological intervention (ECCG II) in 64 (86%) of the patients. One patient (1%) underwent thoracotomy for debridement of the chest cavity (ECCG III). No patient received surgical intervention aimed directly at closing the AL.

Sixty-six patients (89%) had successful closure of the AL after median 27 days (4–127 days). Eleven patients (15%) developed tracheo- or bronchoesophageal airway fistulas, diagnosed at a median of 39 days (15–1927 days) after surgery and a median of 36 days (1–1882 days) after the leakage was diagnosed. The airway fistula healed in five patients by means of a stent (*n* = 2), ethanol injection (*n* = 1), a combination of stent and gluing of the fistula tract (*n* = 1) and spontaneously in one patient. Among those six patients with remaining airway fistula tract, four underwent extensive surgery and two were permanently stented. Patients with airway fistula had more severe leaks, classified according to ECCG, than patients that did not develop fistula (*P* = 0.03). No other differences in patient characteristics, including the use of neoadjuvant (radio)chemotherapy, time of AL diagnosis, nor the type of treatment modality among patients with (*n* = 11) or without airway fistula (*n* = 63) were found (Table 3). In particularly, we did not find any association between treatment with EVT and development of airway fistula.

**Table 3:** Patient characteristics and treatment in patients with (*n* = 11) or without (*n* = 63) airway fistula

**Characteristics**	**With airway fistula (*n* = 11)**	**Without airway fistula (*n* = 63)**
Age (median)	65 years	66 years (*P* = 0.55)
Sex (men)	9 (82%)	54 (86%, *P* = 0.73
BMI	29,7	27 (*P* = 0.19)
ASA (median)	3	2 (*P* = 0.73)
COPD	1 (9%)	12 (19%, *P* = 0.42)
Frailty score (mean)	3	2,65 (*P* = 0.10)
Diabetes	3 (27%)	9 (14%, *P* = 0.27)
Smoking	3 (27%)	26 (42%, *P* = 0.34)
HMIE	0	4 (6%, *P* = 0.40)
TMIE	11 (100%)	60 (93%, *P* = 0.36)
ECCG classification of AL (mean)	2,09	**1,85 (*P* = 0.03)**
Preoperative (radio)chemotherapy	5 (45%)	20 (32%, *P* = 0.40)
Postop day of leak diagnosis (median)	9 (range: 2–45)	8 (range: 1–78, *P* = 0.78)
Postop day of fistula diagnosis (median)	39 (range: 15–1927)	an
FEV1 (% Predicted)	94%	86% (*P* = 0.71)
DLCO (% Predicted)	91%	79% (*P* = 0.33)
Stent	6 (55%)	34 (54%, *P* = 0.95)
Stent + EVT	4 (36%)	10 (16%, *P* = 0.12)
EVT	0	6 (10%, *P* = 0.27)
By other endoscopic means	1 (9%)	1 (2%, *P* = 0.21)
Conservative	0	12 (19%, *P* = 0.11)
<T4	11 (100%)	59 (94%, *P* = 0.40)
T4a/b	0	4 (6%, *P* = 0.40)

ECCG , Esophageal Complications Consensus group; FEV1 , forced expiratory volume during the first second; DLCO , diffusing capacity of the lungs for carbon monoxide; EVT , endoscopic vacuum therapy; Values in bold are statistical significant (*p* < 0.05).

### Treatment resources

The median hospital and ICU stays were 30 (range: 12–285) and 9 (range: 0–60) days, respectively (Table 4). Patients underwent four (0–23) CT-examinations and four (0–62) gastroscopies each. Sixty-three patients (85%) had a CD grade IIIa or more advanced complication, which in 67% of the cases was related to pneumonia. During the first 12 months, 27 patients (36%) had one or more readmissions. One patient had 13 readmissions, mostly due to repetitive endoscopic treatments of an airway fistula. Within 12 months post-esophagectomy, 24 patients (32%) developed a symptomatic esophageal anastomotic stricture in need of treatment.

**Table 4:** Resource usage, treatment outcome, and complications in 74 patients treated for AL after Ivor Lewis esophagectomy during 2010–2021

	**Treatment resources, results and complications**					
	**All patients (*n* = 74)**	**Stent (*n* = 40)**	**EVT (*n* = 6)**	**EVT + stent (*n* = 14)**	**Conservative (*n* = 12)**	**Other (*n* = 2)**
**Treatment resources**	4	4	2 (*P* = 0.13)	7 (*P* = 0.09)	**2 (*P*** **= 0.01)**	4,5
No of CT examinations (median)						
No of gastroscopies (median)	4	3,5	3,5 (*P* = 0.58)	9,5 (*P* = 0.18)	1 (*P* = 0.10)	12
Length of stay ICU (median)	9	9	7 (*P* = 0.27)	18 (*P* = 0.77)	6 (*P* = 0.12)	14,5
Length of stay OUS (median)	30	30,5	28 (*P* = 0.49)	43 (*P* = 0.94)	23 (*P* = 0.14)	49
**Results**						
No of patients achieving leak closure	66 (89%)	34 (85%)	6 (100%, *P* = 0.31)	13 (93%, *P* = 0.44)	12 (100%, *P* = 0.15)	1 (50%)
Median duration of treatment (days)	27	29,5	9,5 (*P* = 0.05)	**55 (*P* = 0.04)**	**7 (*P* < 0.01)**	40
No of patients developing airways fistula	11 (15%)	6 (15%)	0 (*P* = 0.31)	4 (29%, *P* = 0.25)	0 (*P* = 0.15)	1 (50%)
Stricture formation within 12 months	24 (32%)	15 (35%)	2 (33%, *P* = 0.81)	4 (29%, *P* = 0.55)	3 (25%, *P* = 0.41)	0
**Complications**						
*Pneumonia*	42 (57%)	25 (63%)	5 (83%, *P* = 0.33)	5 (36%, *P* = 0.09)	5 (42%, *P* = 0.21)	2 (100%)
*Cardiovascular*	24 (33%)	13 (33%)	1 (17%, *P* = 0.44)	6 (43%, *P* = 0.48)	4 (33%, *P* = 0.97)	0
Necrosis of conduit	4 (9%)	3 (8%)	0 (*P* = 0.48)	1 (7%, *P* = 0.95)	0 (*P* = 0.33)	0
Chyle leak	2 (3%)	2 (5%)	0 (*P* = 0.58)	0 (*P* = 0.39)	0 (*P* = 0.43)	0
No of patients having a serious complications (CD ≥3)	63 (85%)	40 (100%)	6 (100%, *P* = 1)	14 (100%, *P* = 1)	**1 (8%, *P* < 0.01)**	2 (100%)

EVT, endoscopic vacuum therapy; OUS, Oslo University Hospital; CT , computer tomography; Values in bold are statistical significant (*p* < 0.05).

The 30- and 90-days mortality among patients with AL was 4% (*n* = 3) and 5% (*n* = 4), respectively. At median 30.5 (1–103) months postesophagectomy, 39 patients were dead (53%). Twenty-four out of the 39 patients (62%) died due to cancer, 5 (13%) had complications related to AL (failure to rescue), while 10 patients (26%) died related to other causes.

Patients in the ‘conservative group’ underwent significantly fewer CT examinations, had shorter duration of treatment and less complications than the ‘stent group’. Patients in the ‘EVT + stent’ had longer treatment. Table 4 further subclassifies the success of leakage closure, development of airway fistula, symptomatic strictures, hospital resources used, as well as all severe complications (CD $\ge$ IIIa).

## DISCUSSION

The present study underlines that most AL after Ivor Lewis esophagectomy may successfully be treated non-operatively, with conservative or endoscopic measures, including percutaneous drainage of intrathoracic fluid collections or abscesses. All but one patient was treated non-operatively. In other series, up to >20% of patients with AL are reported to be treated surgically.^13^ Time to AL healing was about 1 month and may depend on treatment strategy. The high rate of concomitant airway fistulation observed (15%) complicates treatment and is resource demanding.

The severity of the AL was not classified using a validated scoring system. Based on data from the TENTACLE study, Ubels *et al*. recently described 12 different predictors for severity scoring and together they make up the SEAL score.^14^ By combining different predictors, the leak severity is divided into four groups: mild, moderate, severe, and critical. More severe leaks were associated with increased duration of ICU stay, healing time, higher ECCG grade, and postoperative complications.^14^

The treatment of AL has shifted from extensive surgery to treatment by conservative and endoscopic measures. However, surgery remains an important bail-out strategy for failed treatments by less invasive interventions. As of today, many surgeons treat asymptomatic or minimally symptomatic leaks conservatively, while septic patients or early postoperative leaks may be considered for endoscopic therapy or surgery. In a meta-analysis including 338 patients, EVT had a significant higher closure rate of 85% compared to 65% after SEMS and with 11.6 days shorter time to healing.^15^ The duration of hospital and ICU stay, treatment-related complications, major complications, or rate of esophago-tracheal airway fistulas were comparable, but a lower stricture rate was found in the EVT group. However, the difference in time to healing could be related to imprecise definition of closure rates, since a stent is left in place for several weeks, while in EVT, the perforation is evaluated every 3–5 days. These findings of the meta-analysis are in line with our results, with a tendency of shorter time to healing in the EVT group compared to the stent group (9.5 days vs. 29.5 days, *P* = 0.05) and no difference in hospital or ICU stay, rate of complications, or formation of esophago-tracheal fistulas. However, we did not see any difference in the number of patients achieving AL closure between the groups, nor the stricture formation rates, which could be related to restricted number of patients.

We found longer treatment time in the EVT + stent group and shorter among those treated conservatively, probably owing to the severity of the leakage, where the more advanced cases were selected for the combination treatment. In our practice, there was a trend towards increasing use of EVT alone or in combination with stent (Fig. 2). Our current practice is typically initial EVT with subsequent stenting. The peak rate of AL during 2017–18 was not related to changes in perioperative oncologic treatment, ERP, surgical protocols, patient characteristics including pTNM or ASA classification.

Timing of the diagnosis of AL may be of relevance to therapeutic efficiency and algorithm. In our series, the diagnosis was established at postoperative day 8. Currently, all our patients undergo routine gastroesophageal contrast examinations at day 3 (esophagogram); this was not the routine throughout the study period. Identifying patients at risk for AL, by measures such as intraoperative Indocyanine Green, could allow for earlier or potential prophylactic interventions, but it was not used routinely in our series.^16^

We experienced an airway fistula rate of 15% (11 patients) in patients with AL and 2.1% among all patients operated with Ivor Lewis esophagectomy during the study period. Our data show an association between the severity of the leak, according to ECCG, and development of an airway fistula (*P* = 0.03). We did not find any other variables that predicted development of airway fistula nor any association between formation of airway fistula and treatment modality of AL, specifically not EVT. However, only four patients with airway fistula were treated with EVT, and any potential association needs to be explored in future appropriately sized studies. Fistula rates following AL have been reported between 1–9%.^7^^,^^15^ However, the series are difficult to compare, as the numbers are small and they include different types of esophagectomies and AL-treatment. From 1997 to 2016, we had a total of four airways fistulas (2%) following 232 esophagectomies, which are in line with the literature.^17–19^ During the second half of the study period, we introduced several adjustments to the treatment approach, including TMIE, change from side to prone position during the thoracic part of the procedure, ERP, and the use of EVT. Potential impact of these changes to the high rate of fistulas in patients with AL warrants further exploration.

The therapeutic landscape for handling of AL after esophagectomy is developing. Novel endoscopic techniques include over the scope clips, sealant (e.g. fibrin glue), and endoscopic suturing. Only two patients in our series were treated using these endoscopic modalities, which reflects the scant literature with only small series published and limited documentation of its efficiency and safety.^20^ Promising results from combined and integrated EVT and stent treatment have recently been reported in the context of esophageal AL.^21^

In our series, 18% of the patients had chronic obstructive pulmonary disease (COPD) and the mean FEV1% was 67.5%. A European multicenter study has shown that cardiorespiratory comorbidity is associated with increased risk of postoperative complications, including AL.^22^ More specifically, a FEV > 70% was associated with a decreased risk of complications.

Within 12 months of esophagectomy, 32% of our patients developed a symptomatic esophagogastric anastomotic stricture in need of treatment. AL is a well-known risk factor for the development of strictures, as shown in one observational study of 737 patients undergoing transthoracic or transhiatal esophagectomy, where 13% of all strictures were related to AL.^23^

The costs directly related to EVT and SEMS are relatively low.^24^ However, in one report, the total cost following AL was 33.685 USD for patients treated with stent and 46.136 USD for those treated with EVT, primarily owing to ICU stay.^24^ We did not calculate the hospital costs, but our patients had shorter ICU stay (median 9 days vs. 18 days) than in the paper from Eichelmann *et al*. In addition, we found a decline in ICU stay for each patient during the study period, with a mean stay of about 6.3 days/patient in 2011–2012 and 0.6 days/patient in 2019–2020. Most treatment interventions in our series, including endoscopic procedures, were handled without the need for operating theater resources. We also used a well-functioning homemade EVT system. These factors both reduced hospital costs and the need for resources.

Failure to rescue, i.e. the number of patients who died due to AL, was 13% in our series and in line with other studies.^25^ Low failure to rescue rates have been associated with high-volume centers (> 60 esophagectomies/year), lower leak severity, less secondary ICU readmissions, and higher availability of therapeutic modalities.

This report has several limitations, being a retrospective, single center study with a limited number of patients. Besides a higher proportion of ECCG type I leakages in the conservative treated group, we did not find any differences in leak severity, according to ECCG, across the groups (Table 2). However, our findings are based on patient outcomes according to treatment strategy, not leak severity. Specifically, EVT was more frequently used in patients with high degree of periesophageal contamination, while patients with minor leaks were more often treated with antibiotics and tube, only. Thus, leak severity most likely influenced treatment strategy and hence patient outcomes. The treatment approach varied during the study period. EVT was introduced at our hospital in 2015; thus, only patients in the last part of the study period were able to receive this treatment.

The optimal treatment of AL is yet to be decided. However, in our experience, for most patients, the gastric conduit can be preserved and the leakage can been treated endoscopically. Except for minor leakages in clinical stable patients, we typically initiate the treatment with EVT and percutaneous drainage of any mediastinal and/or pleural fluid collections/abscesses. After infection control and a few EVT changes, we place a fully covered SEMS for approximately 3 weeks. This non-operative approach demands that the conduit is non-necrotic, the anastomosis has not separated completely, and in case of empyema, that the patient responds to non-surgical measurements.

In summary, 89% of all AL following Ivor Lewis esophagectomy healed without surgical treatment. All but one patient was treated non-operatively. Further studies should focus on optimal treatment strategies to ensure high rates of healing and low rates of complications associated with esophageal AL.
